# Antiapoptotic effects of cannabidiol in an experimental model of cognitive decline induced by brain iron overload

**DOI:** 10.1038/s41398-018-0232-5

**Published:** 2018-09-03

**Authors:** Vanessa Kappel da Silva, Betânia Souza de Freitas, Rebeca Carvalho Lacerda Garcia, Ricardo Tavares Monteiro, Jaime Eduardo Hallak, Antônio Waldo Zuardi, José Alexandre S. Crippa, Nadja Schröder

**Affiliations:** 1 Neurobiology and Developmental Biology Laboratory, Faculty of Biosciences, Pontifical Catholic University of Rio Grande do Sul, Porto Alegre, 90619-900 Brazil; 2 National Institute of Science and Technology for Translational Medicine (INCT-TM), Conselho Nacional de Desenvolvimento Cientifico e Tecnologico (CNPq), Brasília, Brazil; 3 Department of Neuroscience and Behavior, Ribeirão Preto Medical School, University of São Paulo, São Paulo, 14048-900 Brazil; 4 Departamento de Fisiologia, Universidade Federal do Rio Grande do Sul, Porto Alegre, 90050-170 Brazil

## Abstract

Iron accumulation in the brain has been recognized as a common feature of both normal aging and neurodegenerative diseases. Cognitive dysfunction has been associated to iron excess in brain regions in humans. We have previously described that iron overload leads to severe memory deficits, including spatial, recognition, and emotional memory impairments in adult rats. In the present study we investigated the effects of neonatal iron overload on proteins involved in apoptotic pathways, such as Caspase 8, Caspase 9, Caspase 3, Cytochrome c, APAF1, and PARP in the hippocampus of adult rats, in an attempt to establish a causative role of iron excess on cell death in the nervous system, leading to memory dysfunction. Cannabidiol (CBD), the main non-psychotropic component of *Cannabis sativa*, was examined as a potential drug to reverse iron-induced effects on the parameters analyzed. Male rats received vehicle or iron carbonyl (30 mg/kg) from the 12th to the 14th postnatal days and were treated with vehicle or CBD (10 mg/kg) for 14 days in adulthood. Iron increased Caspase 9, Cytochrome c, APAF1, Caspase 3 and cleaved PARP, without affecting cleaved Caspase 8 levels. CBD reversed iron-induced effects, recovering apoptotic proteins Caspase 9, APAF1, Caspase 3 and cleaved PARP to the levels found in controls. These results suggest that iron can trigger cell death pathways by inducing intrinsic apoptotic proteins. The reversal of iron-induced effects by CBD indicates that it has neuroprotective potential through its anti-apoptotic action.

## Introduction

Iron accumulation has been described in normal ageing in several brain regions and cell types. However, in neurological disorders such as Alzheimer’s (AD), Parkinson’s (PD), and Huntington’s (HD) diseases, iron accumulates in selective brain areas such as the hippocampus, substantia nigra, cortex, and basal ganglia, regions relevant to disease-associated neurodegenerative processes^1–3^.

The exact mechanisms that underlie neurotoxicity induced by iron and other metals are not completely understood. In previous studies, we have established an animal model of brain iron loading, with oral administration of iron during the neonatal phase, period of maximal iron uptake by the brain^4^ to better characterize the effects of iron excess on brain function. We have previously described that iron overload induces severe and persistent long-term impairments in spatial, recognition, and emotional memories^5–11^. In molecular analyses, we found lipid peroxidation and oxidative damage associated with iron excess^6^, increased apoptotic markers, Par4^12^ and Caspase 3^13^, accumulation of ubiquitinated proteins^14^, and reactive gliosis^15^. Moreover, iron treatment in the neonatal period decreased acetylcholinesterase activity in the striatum^8^ and affected the regulation of iron homeostasis proteins in the hippocampus, cortex, and striatum of aged rats^16^. In addition, there was a decrease in synaptophysin levels, a marker of synaptic viability, and changes in DNM1L levels, a protein critically involved in mitochondrial dynamics in the hippocampus of iron-treated rats^13^. We have also demonstrated that iron chelation prevented memory impairments and oxidative stress in aged rats, supporting the concept that cognitive deficits associated with aging might be related to iron accumulation in the brain^17^.

Apoptosis is a major form of programmed cell death that has been implicated in neurodegenerative disorders^18^. Studies have consistently reported deregulations in the expression of apoptotic proteins in the brains of both PD and AD patients and in experimental models of neurodegenerative disorders (for a review, see^19,20^). Among the stimuli known to trigger apoptosis are alterations of the redox balance and oxidative damage^21,22^.

Cannabidiol (CBD) is a compound currently being investigated as a potential therapeutic option for neurodegenerative disorders. CBD is the main non-psychotropic constituent of *Cannabis sativa*, corresponding to about 40% of the plant extract^23^. Evidence indicates that CBD has antioxidant, antiapoptotic, and neuroprotective properties^24–28^ (reviewed in ref. ^29^). Our previous studies showed that CBD is able to improve iron-induced memory deficits^10^ and regulate markers of synaptic viability and mitochondrial dynamics in the hippocampus of iron-overloaded rats^13^.

The aim of the present study was to characterize the effects of iron loading on proteins critically involved with apoptotic processes in the hippocampal formation. We first examined Caspase 3, which is a caspase of the final common pathway of apoptosis, activated both by extrinsic and intrinsic apoptotic pathways, and cleaved PARP, one of several known cellular substrates cleaved by Caspase 3. Then we analyzed Caspase 8 and Caspase 9, initiator caspases to extrinsic and intrinsic apoptotic pathways, respectively. In order to confirm the involvement of the intrinsic apoptotic pathway, we investigated the effects of excessive iron on Cytochrome c, a protein released when mitochondrial membrane permeability is altered, involved in apoptosis initiation, and APAF1, an adapter protein responsible for the formation of apoptosome and activation of pro-caspases in the intrinsic pathway. Considering that CBD was able to restore memory in iron-treated rats, we examined possible neuroprotective effects of CBD against iron-induced deregulation of apoptotic players.

## Material and methods

### Animals

Pregnant Wistar rats (3 months old) (CrlCembe:WI) were obtained from the *Centro de Modelos Biológicos Experimentais* (CeMBE) of the Pontifical Catholic University in Porto Alegre, RS, Brazil. After birth, each litter was adjusted within 48 h to eight rat pups including offspring of both genders in about equal proportions and kept at standard laboratory conditions. At the age of three weeks, pups were weaned and the males were selected and raised in groups of three to five in individually ventilated cages with sawdust bedding. For postnatal treatments, animals were given standardized pellet food and tap water ad libitum.

All experimental procedures were performed in accordance with the Brazilian Guidelines for the Care and Use of Animals in Research and Teaching (DBCA, published by CONCEA, MCTI, Brazil) and approved by the Institutional Ethics Committee for the Use of Animals of the Pontifical Catholic University (CEUA 14/00409). All efforts were made to minimize the number of animals and their suffering.

### Treatments

#### Neonatal iron treatment

The neonatal iron treatment has been described in detail elsewhere^10,13^. Briefly, 12-day-old male rat pups from randomly assigned litters (simple randomization) received a single oral daily dose of vehicle (5% sorbitol in water, control group) or 30 mg/kg of body weight of Fe^2+^ (iron carbonyl, Sigma-Aldrich, São Paulo, Brazil) via a metallic gastric tube, over 3 days (postnatal days 12–14).

#### Cannabidiol

Adult (3-month-old) rats, treated neonatally with either vehicle or iron as described above, received a daily intraperitoneal injection of vehicle (Tween 80 – saline solution 1:16 v/v) or CBD (10 mg/kg, ~99.9% pure; kindly supplied by BSPG-Pharm, Sandwich, UK) for 14 consecutive days. Dose and duration of treatment were chosen based on previously published articles from our research group, showing that CBD was able to completely reverse iron-induced memory deficits^10^ and synaptic alterations^13^. Drug solutions were freshly prepared immediately prior to administration^10,13^.

Rats were euthanized by decapitation at 24 h after the last injection of CBD. Brains were quickly dissected and hippocampi were isolated and stored at −80 °C for subsequent Western Blotting or enzyme-linked immunosorbent (ELISA) assays. Sample size estimation was based on previously published papers from our research group reporting iron and CBD effects on biochemical parameters measured using western blot and ELISA^13,30^.

### Molecular analyses

#### Western blotting analysis

Proteins were extracted as previously described by da Silva and coworkers^13^. The supernatant was collected and the protein content was determined using Bradford assay^31^. Aliquots were stored at − 20 °C.

Fifty (50) µg of protein were separated on a 10% SDS polyacrylamide gel and transferred electrophoretically to a nitrocellulose membrane. Membranes were blocked with 5% albumin in TBS containing 0.05% Tween 20 and incubated overnight with one of the following antibodies: anti-Caspase 3 (ab44976, Abcam, Cambridge, UK) at 1:500; anti-Caspase 9 (ab2013, Abcam, Cambridge, UK) at 1:500; anti-PARP (ab6079, Abcam, Cambridge, UK) at 1:200; anti-Cleaved-Caspase 8 (9429, Cell Signaling, Danvers, USA) at 1:600 and anti-Tubulin (ab52866, Abcam, Cambridge, UK) at 1:20000. Goat polyclonal anti-rabbit IgG H&L (HPR) (ab6721, Abcam, Cambridge, UK) secondary antibody was used and detected using ECL western blotting Substrate Kit (ab65628, Abcam, Cambridge, UK). Pre-stained molecular weight protein markers (84785, SuperSignal Molecular Weight Protein Ladder, Thermo Scientific, Rockford, USA) were used to determine the detected bands molecular weight and confirm antibodies target specificity. The densitometric quantification was performed using Chemiluminescent photo finder (Kodak/Carestream, model GL2200) by an experimenter blind to samples experimental condition. Total blotting protein levels of samples were normalized according to each sample’s Tubulin protein levels (adapted from ref. ^13^).

#### Enzyme-linked immunosorbent assay (ELISA)

We used sandwich-ELISA commercial kits to measure hippocampal APAF1 (LS-F8140, LSBio, Seattle, USA) and Cytochrome c proteins (LS-F11266, LSBio, Seattle, USA). Briefly, hippocampi were finely minced and homogenized in 750 µL of PBS with a glass homogenizer on ice. Cells were lysed by 3 cycles of freeze (−20 °C) / thaw (room temperature). Homogenates were centrifuged at 5000 × g for 5 min and the supernatant was collected for assaying. One hundred µL of standard, blank and samples were tested in duplicate and the optical density was determined using a microplate reader set to 450 nm. The standard curve demonstrated a direct relationship between optical density and APAF1 or Cytochrome c concentrations. Results were expressed as nanograms of APAF1 or Cytochrome c per μg of protein obtained from tissue homogenates. Total protein was measured by Bradford’s method using bovine serum albumin as protein standard^31^.

### Statistical analysis

The results were analyzed using SPSS 20.0 and expressed as means ± S.E.M. Levene’s test of equality of variances was used in order to test the assumption of homogeneity of variance. Variances were similar among the experimental groups for all tested variables. Statistical comparisons were performed using two-way analysis of variance (2-way-ANOVA), with neonatal treatment (vehicle or iron) and adult treatment (vehicle or CBD) as fixed factors. One-way ANOVA, followed by Tukey’s post hoc test, was used to test differences between the experimental groups. In all comparisons, *p* values below 0.05 were considered as indicative of statistical significance.

## Results

**Fig. 1:**
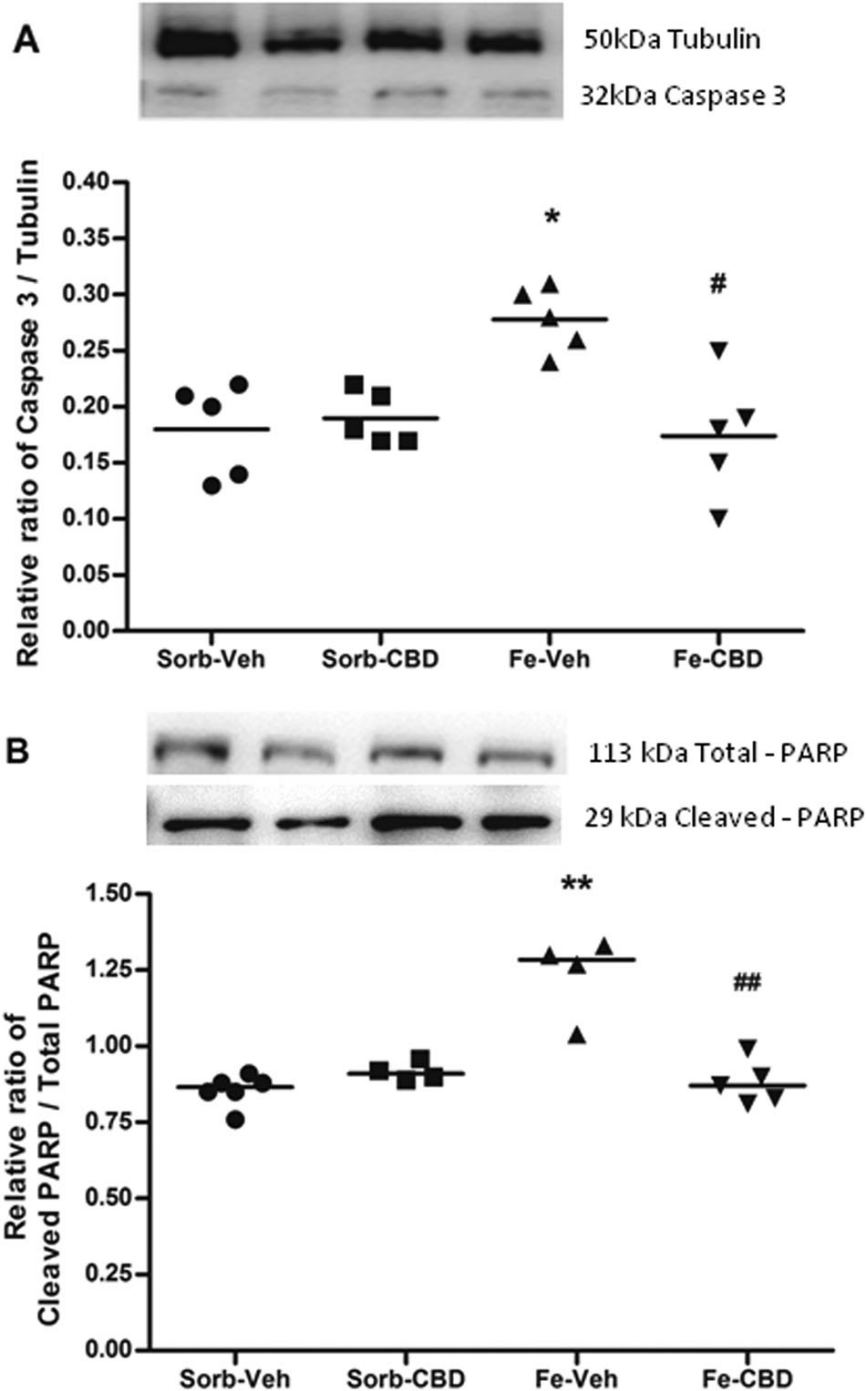
Iron treatment increases and CBD restores Caspase 3 and cleaved-PARP levels. **a** Western Blotting of Caspase 3 (*N* = 5 rats per group) and (**b**) ratio of cleaved PARP to total PARP (Sorb-Veh *N* = 6, Sorb-CBD *N* = 4, Fe-Veh *N* = 4, Fe-CBD *N* = 5 rats per group) in the hippocampus of rats treated neonatally with sorbitol or iron and treated with vehicle or CBD chronically in the adulthood (3 months of age). Fifty µg of protein were separated on SDS-PAGE and probed with specific antibodies, normalized to Tubulin. Representative western blots for Caspase 3, cleaved PARP, total PARP and Tubulin are shown in the upper panel. Individual data points are plotted and mean values are presented. Statistical analysis was performed using two-way ANOVA and subsequently one-way ANOVA followed by Tukey HSD post hoc test. *p < 0.01 differences between sorbitol-vehicle (Sorb-Veh) vs. iron-vehicle (Fe-Veh); **p < 0.0001 differences between Sorb-Veh vs. Fe-Veh. #*p* < 0.01 difference between Fe-Veh vs. iron-CBD (Fe-CBD); ##*p* < 0.0001 difference between iron-vehicle vs. iron-CBD

Aiming to evaluate the effects of iron loading in the neonatal period on apoptosis, we first examined the effects of iron treatment on Caspase 3 and cleaved PARP. Statistical comparisons of Caspase 3 levels, measured by western blot, using 2-way ANOVA indicated a significant main effect of neonatal treatment (F_(1,16)_ = 5.47, *p* < 0.05), a significant main effect of adult treatment (F_(1,16)_ = 7.18, *p* < 0.05, Fig. 1a), and a significant interaction (F_(1, 16)_ = 10.57, *p* < 0.01, Fig. 1a). Further comparisons using one-way ANOVA revealed significant differences among the groups (F_(3,16)_ = 7.74, *p* < 0.01, Fig. 1a). Post hoc comparisons between groups indicated that neonatal iron treatment significantly increased Caspase 3 in comparison to the control group (*p* < 0.01). CBD was able to completely reverse iron-induced effects on Caspase 3, since measures from the iron-CBD group were statistically different from those of the iron-vehicle group (*p* < 0.01), but not from the control group (*p* = 0.995). In order to confirm the activation of Caspase 3, we quantified the ratio of cleaved PARP to total PARP. Two-way ANOVA revealed a significant main effect of neonatal treatment (F_(1,15)_ = 22.88, *p* < 0.0001), a significant main effect of adult treatment (F_(1,15)_ = 16.69, *p* < 0.01), and a significant interaction (F_(1,15)_ = 34.00, *p* < 0.0001, Fig. 1b). Comparisons using one-way ANOVA revealed a significant difference among the groups (F_(3,15)_ = 22.96, *p* < 0.0001, Fig. 1b). Neonatal iron treatment significantly increased cleaved PARP in comparison to the control group (*p* < 0.0001), while CBD treatment in adulthood reversed this effect. The iron-CBD group presented significantly lower cleaved PARP in relation to total PARP than the iron-vehicle group (*p* < 0.0001) and was not significantly different from the control group (*p* = 0.949).

**Fig. 2:**
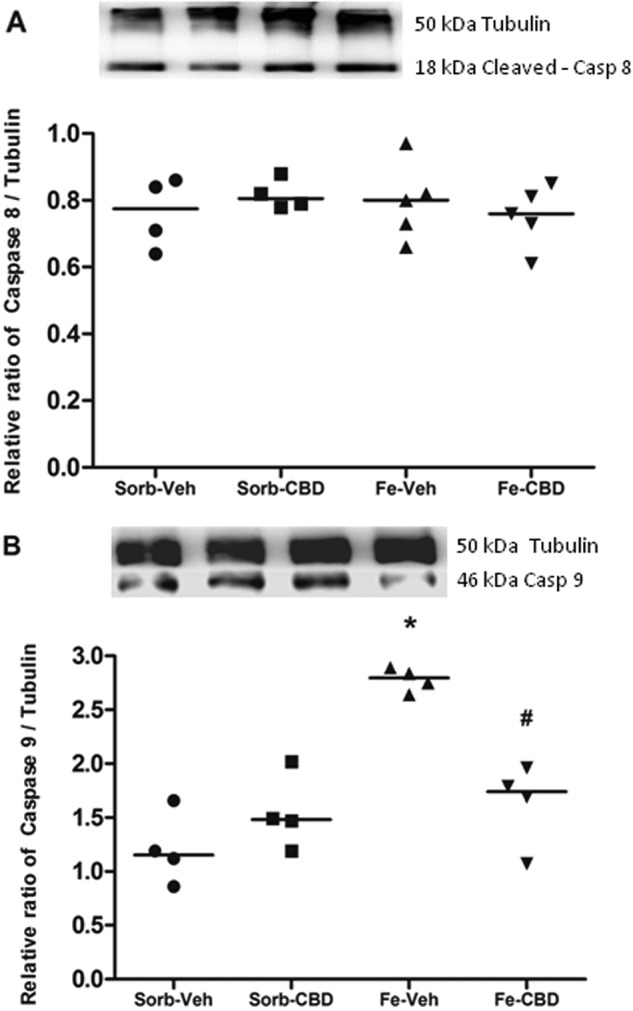
Iron treatment increases and CBD restores Caspase 9 levels, without affecting cleaved-Caspase 8. **a** Western Blotting of cleaved-Caspase 8 (Sorb-Veh *N* = 4, Sorb-CBD *N* = 4, Fe-Veh *N* = 5, Fe-CBD *N* = 5 rats per group) and (**b**) Caspase 9 (*N* = 4 rats per group) in the hippocampus of rats treated neonatally with sorbitol or iron and treated with vehicle or CBD chronically in the adulthood (3 months of age). Fifty µg of protein were separated on SDS-PAGE and probed with specific antibodies and normalized to Tubulin. Representative Western Blots for cleaved-Caspase 8, Caspase 9 and Tubulin are shown in the upper panel. Individual data points are plotted and mean values are presented. Statistical analysis was performed using two-way ANOVA and subsequently one-way ANOVA followed by Tukey HSD post hoc test. *p < 0.0001 indicates a significant increase in Caspase 9 protein expression in the iron-vehicle (Fe-Veh) group compared to controls. #*p* ≤ 0.001 indicates a significant decrease in Caspase 9 protein expression in the iron-CBD (Fe-CBD) group compared to Fe-Veh

We also analyzed the effects of neonatal iron loading and adult treatment with CBD on Caspases 8 and 9. Two-way ANOVA comparisons of cleaved-Caspase 8 measured by western blot revealed no significant main effects of neonatal treatment (F_(1,14)_ = 0.13, *p* = 0.728, Fig. 2a) or adult treatment (F_(1,14)_ = 0.015, *p* = 0.905, Fig. 2a). However, statistical comparisons of Caspase 9 levels using two-way ANOVA showed significant main effects of neonatal treatment (F_(1,12)_ = 27.90, *p* < 0.0001, Fig. 2b) and adult treatment (F_(1,12)_ = 6.79, *p* < 0.05, Fig. 2b), and a significant interaction (F_(1,12)_ = 22.47, *p* < 0.0001, Fig. 2b). One-way ANOVA revealed significant differences in Caspase 9 protein levels among the groups (F_(3, 12)_ = 19.05, *p* < 0.0001, Fig. 2b). Post hoc comparisons between groups indicated that neonatal iron treatment induced a significant increase in Caspase 9 protein levels in comparison to the control group, which received sorbitol in the neonatal period and vehicle in adulthood (*p* < 0.0001). The reversion effects of CBD were also observed, considering that the group that received iron in the neonatal period and CBD in adulthood (iron-CBD) showed statistically significant differences in Caspase 9 levels in comparison to the group that received iron in the neonatal period and vehicle in adult age (*p* = 0.001), and this group was not significantly different from the control group (*p* = 0.281).

**Fig. 3:**
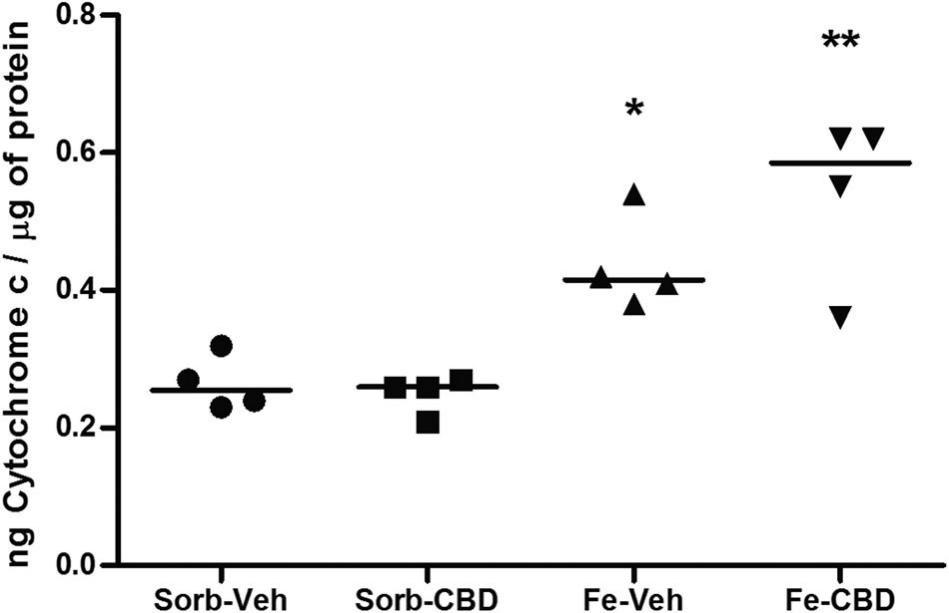
Iron treatment increases Cytochrome c levels. Cytochrome c protein levels measured by ELISA assay in the hippocampus of rats treated neonatally with sorbitol or iron and treated with vehicle or CBD chronically in the adulthood (3 months of age). Statistical analysis was performed using two-way ANOVA and subsequently one-way ANOVA followed by Tukey HSD post hoc. Individual data points are plotted and mean values are presented. *N* = 4 rats per group. *p < 0.05 indicates a significant increase in Cytochrome c protein expression in the iron-vehicle (Fe-Veh) group compared to controls. **p ≤ 0.001 indicates a significant increase in Cytochrome c protein expression in the iron-CBD (Fe-CBD) group compared to controls

In order to confirm the involvement of the intrinsic apoptotic pathway, we then decided to investigate the effects of excessive iron on Cytochrome c levels. Two-way ANOVA revealed a significant main effect of neonatal treatment (F_(1,12)_ = 37.76, *p* < 0.0001, Fig. 3), but no significant main effect of adult treatment (F_(1, 12)_ = 1.29, *p* = 0.278, Fig. 3) nor interaction (F_(1, 12)_ = 2.36, *p* = 0.15, Fig. 3) were observed. One-way ANOVA comparisons of Cytochrome c levels demonstrated a significant difference among the groups (F_(3,12)_ = 13.80, *p* < 0.0001, Fig. 3). When groups were compared using Tukey’s post hoc test, results revealed that neonatal iron treatment induced a significant increase in Cytochrome c levels when compared to the control group (Sorb-Veh, *p* < 0.05). The iron-treated group that received CBD in adulthood had also significantly higher Cytochrome c levels when compared to the control group (*p* = 0.001), suggesting that CBD was not able to reverse the effects of iron loading on Cytochrome c protein levels.

**Fig. 4:**
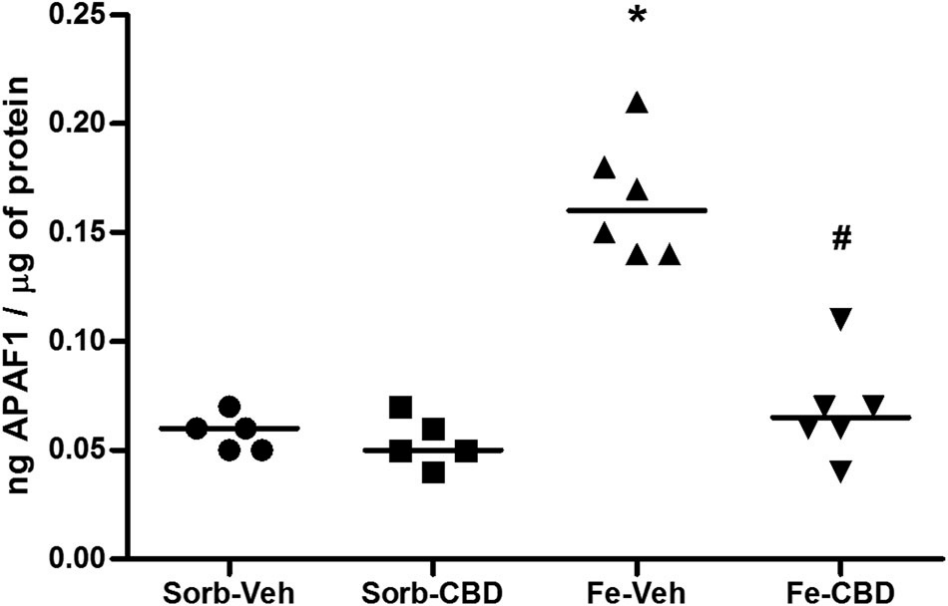
Iron treatment increases and CBD restores APAF1 levels. APAF1 protein levels measured by ELISA assay in the hippocampus of rats treated neonatally with sorbitol or iron and treated with vehicle or CBD chronically in the adulthood (3 months of age). Individual data points are plotted and mean values are represented. Sorb-Veh *N* = 5, Sorb-CBD *N* = 5, Fe-Veh *N* = 6, Fe-CBD *N* = 6 rats per group. Statistical analysis was performed using two-way ANOVA and subsequently one-way ANOVA followed by Tukey HSD post hoc test. *p < 0.0001 indicates a significant increase in APAF1 protein expression in the iron-vehicle (Fe-Veh) group compared to controls. #*p* < 0.0001, indicates a significant decrease in APAF1 protein expression in the iron-CBD (Fe-CBD) group compared to Fe-Veh

We also aimed to investigate the long-term consequences of neonatal iron loading and adult treatment with CBD on APAF1 protein levels. A statistical comparison of APAF1 levels using 2-way ANOVA revealed a significant main effect of neonatal treatment (F_(1,18)_ = 49.96, *p* < 0.0001, Fig. 4), a significant main effect of adult treatment (F_(1,18)_ = 34.39, *p* < 0.0001, Fig. 4) and a significant interaction (F_(1,18)_ = 29.14, *p* < 0.0001, Fig. 4). One-way ANOVA showed significant differences among the groups (F_(3,18)_ = 39.9, *p* < 0.0001, Fig. 4). Post hoc comparisons between groups indicated that neonatal iron treatment significantly increased APAF1 levels in comparison to the control group, which received sorbitol in the neonatal period and vehicle in adulthood (*p* < 0.0001). The iron-treated group that received CBD in adult age showed statistically significant differences in APAF1 when compared to the group that received vehicle in adult age (*p* < 0.0001) and no significant differences were observed in comparison to the control group (*p* = 0.829), indicating that CBD was able to completely reverse iron-induced effects on APAF1.

## Discussion

The present results showed that neonatal iron-treatment led to significant changes in the concentration of apoptotic proteins, increasing all intrinsic apoptotic pathway proteins analyzed. Iron has the ability to exchange single electrons with many different substrates and, as a result of the participation of iron in Fenton chemistry, this metal can lead to the generation of reactive oxygen species (ROS)^32^. ROS trigger oxidative stress, inducing lipid peroxidation and DNA damage that can lead to impaired cell viability and initiation of signaling pathways crucial for cell survival and cell death^33^.

Aiming to gain a better understanding of the mechanisms involved and trying to establish a possible causative role of iron overload in apoptosis, we have investigated key players in the apoptotic pathway in the hippocampus of adult rats submitted to iron overload in the neonatal period. Previous studies performed by our research group have shown that neonatal iron treatment induces lipid peroxidation and increases mitochondrial superoxide generation in the hippocampus, cortex, and substantia nigra^6,34^ and protein carbonylation in the substantia nigra^35^ in adult rats. In line with the present results, in previous studies we have also observed an increase in the apoptotic markers, Caspase 3^13^ and Par4^12^ in the brains of iron-treated rats. Corroborating the present findings, You and colleagues^36^ found that excess of iron in the substantia nigra increased oxidative stress levels, promoting apoptosis through the Bcl-2 / Bax pathway and the activated Caspase3 pathway in an animal model of PD. In cultures of hippocampal slices exposed to iron, ROS formation and lipid peroxidation were increased, in association with Cytochrome c and Caspase 3-dependent apoptotic pathways^37^. Iron overload in the neonatal period induces severe hippocampus-dependent memory deficits, indicating hippocampal dysfunction^5–9,11,14^, while studies performed in humans have correlated iron accumulation in selective brain regions with poor performance in cognitive tests (for a review, see ref. ^38^). We have evidence that the effects of neonatal iron overload in the brain intensify gradually throughout life. For instance, iron treatment increased the content of ubiquitinated proteins, a marker of UPS-ubiquitin system deterioration, in the hippocampus of adult rats, while no effects were observed when analysis was performed earlier in life^14^. In fact, studies using aged rats and mice suggest that iron accumulates and redistributes in brain regions during life without a coincident increase in ferritin, the main cellular iron storage protein in neurons, suggesting an age-related iron dyshomeostasis^39,40^. Thus, we believe that the deleterious effects of iron overload in the neonatal period will be revealed at later stages in life. In humans, cognitive behavior is influenced by age-associated increases in brain iron content^41,42^. On the basis of these findings, we suggest that iron-induced increased apoptosis later in life might lead to functional deficits observed in our animal model and in patients, implicating iron in the pathogenesis of memory dysfunction associated to aging and neurodegenerative disorders.

The present findings show that iron overload induced increases in APAF1, Caspase 3, Caspase 9, Cytochrome c, and cleaved PARP levels. Although we have not performed a direct measurement of apoptosis, increased cleaved PARP levels have been considered a marker of apoptosis because this protein is the substrate of activated caspases^43^. In agreement, upregulation of Caspase 3 gene expression in a model of cognitive impairment induced by sevoflurane was associated with increased cleaved PARP levels^44^. While Caspase 3 is an effector caspase, being part of the final common pathway of apoptosis, Cytochrome c, APAF 1, and Caspase 9 integrate the intrinsic apoptotic pathway. Interestingly, no alterations in cleaved-Caspase 8 levels were found, confirming that there was no activation of extrinsic apoptosis pathways. On the other hand, we found increases in Caspase 9, Cytochrome c and APAF1 levels, suggesting that the intrinsic pathway is most significantly affected by iron overload. Since mitochondria are the main source of ROS, they are expected to become an important target of oxidative damage, which could explain functional alterations in these organelles in pathological conditions^45^. Moreover, mitochondria play a key role in regulating the intrinsic apoptotic pathway, and there is evidence indicating that iron affects mitochondrial homeostasis^13,30^, thus supporting the concept that iron effects are most pronounced in the intrinsic pathway. Nonetheless, more studies on the effects of iron on the extrinsic pathway are warranted.

Nowadays, many studies are being performed with CBD aiming to analyze its therapeutic properties and mechanisms of action. In this study, we showed the neuroprotective effects of the adult treatment with CBD on apoptotic markers in rats treated neonatally with iron. Considering that iron dyshomeostasis takes place throughout life, and is possibly related to an increased iron intake during early stages of life, CBD might represent a therapeutic option that ameliorates pathological processes previously initiated. We observed that adult treatment with CBD was able to rescue APAF1, Caspase 9, Caspase 3, and cleaved PARP levels. Only Cytochrome c levels were not rescued to control levels. Notwithstanding, taken together the present findings suggest that CBD was able to protect from apoptosis by reducing Caspase 3 and cleaved PARP levels, proteins that participate in the effector phase of apoptosis, which culminates in cell death. A previous study indicated that CBD attenuates the imbalance between Bcl-2/Bax, upregulating the anti-apoptotic protein Bcl-2, which in turn maintains the integrity of the outer mitochondrial membrane, in an animal model of multiple sclerosis^46^. In agreement, we have previously demonstrated that CBD protects against mitochondrial injury^13,30^, converging to prevent the activation of the intrinsic apoptotic pathway. Using a model of HD, Valdeolivas and coworkers^47^ have suggested the involvement of CB1 and CB2 cannabinoid receptors as well as receptor-independent actions in the neuroprotective effects of a CBD-enriched botanical extract. Further studies are warranted in order to clarify whether CBD’s antiapoptotic effects are related or not to cannabinoid receptor agonism.

Although the mechanisms of action of CBD have not been completely elucidated, among the actions proposed for CBD is its antioxidant capacity (see ref. ^48^ for a review). In 2016, Chen and colleagues^49^ found that CBD treatment was able to protect cells in cultures exposed to H_2_O_2_ to generate oxidative stress against apoptotic, inflammatory, and oxidative activities, suggesting that CBD acts by modulating these pathways. Using a mouse model of ischemia, investigators found that CBD attenuated oxidative damage, increased antioxidant defenses, improved mitochondrial function and energetic metabolism, and regulated apoptotic markers in hippocampal neurons^50^. Previously, we studied the effects of CBD in rats submitted to iron overload and observed that CBD recovered mitochondrial dynamic and synaptic viability, besides reducing Caspase 3 in the hippocampus of adult rats^13^. Since we could observe the anti-oxidant, anti-apoptotic, and mitochondrial preservation properties related to neuroprotection, it is clear that no single mechanism will explain the remarkable pharmacological profile of CBD^51^. Therefore, the mechanism of action of CBD must include the modulation of several pathways that, together, improve cellular metabolism and confer neuroprotection, which may account for rescuing the functional deficits observed in our model^10^.

In summary, we have shown that iron treatment in the neonatal period disrupts the apoptotic intrinsic pathway. This finding may place iron excess as a central component in neurodegenerative processes since many neurodegenerative disorders are accompanied by iron accumulation in brain regions. Moreover, indiscriminate iron supplementation to toddlers and infants, modeled here by iron overload in the neonatal period, has been considered a potential environmental risk factor for the development of neurodegenerative disorders later in life^52^. Our findings also strongly suggest that CBD has neuroprotective effects, at least in part by blocking iron-induced apoptosis even at later stages, following iron overload, which puts CBD as a potential therapeutic agent in the treatment of neurodegenerative diseases.

## Disclaimer

The funding sources had no role in the collection, analysis and interpretation of the data, in the writing of the report, and in the decision to submit the paper for publication.
